# The effect of parental pain, disability benefits and education on risk of long-term sick leave due to musculoskeletal disorders and the modifying effect of sleep and physical activity: the HUNT study

**DOI:** 10.1186/s12889-024-20071-1

**Published:** 2024-09-27

**Authors:** Karoline Moe, Eivind Schjelderup Skarpsno, Tom Ivar Lund Nilsen, Paul Jarle Mork, Lene Aasdahl

**Affiliations:** 1 Department of Public Health and Nursing, Faculty of Medicine and Health Sciences, Norwegian University of Science and Technology (NTNU), Postboks 8905, Trondheim, 7491 Norway; 2 Department of Neurology and Clinical Neurophysiology, St. Olavs Hospital, Trondheim, Norway; 3 Unicare Helsefort Rehabilitation Centre, Rissa, Norway

**Keywords:** Sleep, Physical activity, Sick leave, Work disability, Musculoskeletal disorders, Family factors, Young adults, Cohort study

## Abstract

**Background:**

Family factors, sleep, and physical activity have previously been associated with risk of sick leave and disability benefits due to musculoskeletal disorders. However, how these factors act during adolescence and young adulthood is unclear. The aim of this study was to (i) examine if chronic pain, disability benefits and education in parents influence the risk of long-term sick leave due to musculoskeletal disorders in young adulthood, and (ii) to explore if offspring sleep problems and physical activity modify these effects.

**Methods:**

A population-based prospective study of 18,552 adolescents and young adults (≤ 30 years) in the Norwegian HUNT Study. Survey data was linked to national registry data on sick leave, disability benefits, family relations and educational attainment. We used Cox regression to estimate hazard ratio (HR) with 95% confidence interval (CI) for long-term (≥ 31 days) sick leave due to a musculoskeletal disorder in offspring associated with parental factors and the joint effect of parental factors and offspring lifestyle factors.

**Results:**

Parental chronic pain (HR 1.36, 95% CI 1.27–1.45), disability benefits (HR 1.41, 95% CI 1.33–1.48) and low educational attainment (HR 1.78, 95% CI 1.67–1.90) increased the risk of long-term sick leave due to musculoskeletal disorders among offspring. There was no strong evidence that sleep and physical activity modified these effects.

**Conclusion:**

Chronic pain, disability benefit and low education in parents increased the risk of long-term sick leave due to musculoskeletal disorders among offspring, but these effects were not modified by offspring sleep problems or physical activity level. The findings suggest that efforts beyond individual lifestyle factors might be important as preventive measures.

**Supplementary Information:**

The online version contains supplementary material available at 10.1186/s12889-024-20071-1.

## Background

Many young people leave the labor market early due to health complaints [1–3], with vast consequences for both the individuals and the society [1, 3, 4]. A rising number of young individuals transitioning from long-term sick leave benefits to disability benefits is a significant concern in many countries [3]. Musculoskeletal disorders are reported in about 30% of young adults [5], and is the main reason young men visit their general practitioner in Norway [6]. Furthermore, musculoskeletal complaints, together with mental health disorders, dominate sick leave statistics for the working population [1]. While mental disorders are the leading cause of years lived with disability in people < 30 years, the prevalence of musculoskeletal complaints increase with age and are the leading cause in those 25–64 years [7].

Family conditions, such as socioeconomic status in childhood [8] and parents receiving disability benefits [9, 10] have been associated with the risk of receiving permanent disability benefits. Further, studies suggest that musculoskeletal pain and disability benefits due to musculoskeletal disorders have a moderate to strong genetic component [9–13], and that environmental influences on disability benefits are age-specific [9, 10]. Genetic factors may reflect liability to disease, health symptoms, comorbidity, and psychological traits such as pain tolerance and self-efficacy [14, 15]. In a life course perspective, both timing of the exposure and transmission of risk factors across generations may influence the risk of chronic diseases [16, 17] and work-related medical benefits later in life. Assessing vulnerability to long-term sick leave at an individual level may therefore be insufficient, as family conditions and parental characteristics may play an important role.

Previous studies have found that lifestyle factors, such as sleep [18–23] and physical activity [24–28] are associated with risk of sick leave and disability benefits due to musculoskeletal disorders. However, the impact of these exposures acting during adolescence and young adulthood is unclear. It may be hypothesized that the impact of adverse family conditions on later sick leave and risk of disability benefits can be modified by the offspring’s own lifestyle. Insight into these associations could help identify sub-groups in the population that may have additional benefit from lifestyle interventions.

The aim of the current study was twofold. First, to prospectively examine the effect of chronic pain, disability benefits and education in parents on the risk of long-term sick leave due to musculoskeletal disorders in the young adult offspring, and second, to explore if offspring sleep problems or physical activity in adolescence modify these effects.

## Methods

### Study population

This study uses data from the population-based HUNT Study. All inhabitants aged 20 years and above in Nord-Trøndelag County, Norway, were invited to participate in four separate surveys conducted with approximately 11-year intervals between 1984 and 2019 (HUNT1-HUNT4). In the three latest surveys, all adolescents aged 13 to 19 years were also invited to participate (YoungHUNT1-YoungHUNT4). Details of the different surveys are described elsewhere [29, 30]. For those who participated in more than one survey, we used data from their first participation.

The unique personal identification number held by all Norwegian citizens was used to link HUNT data to nationwide registries. A linkage to the Norwegian Labour and Welfare Administration (NAV) obtained data on sick leave and disability benefits, including dates and diagnosis, whereas educational attainment and the family-linkage relating offspring to their parents was obtained from Statistics Norway.

**Fig. 1:**
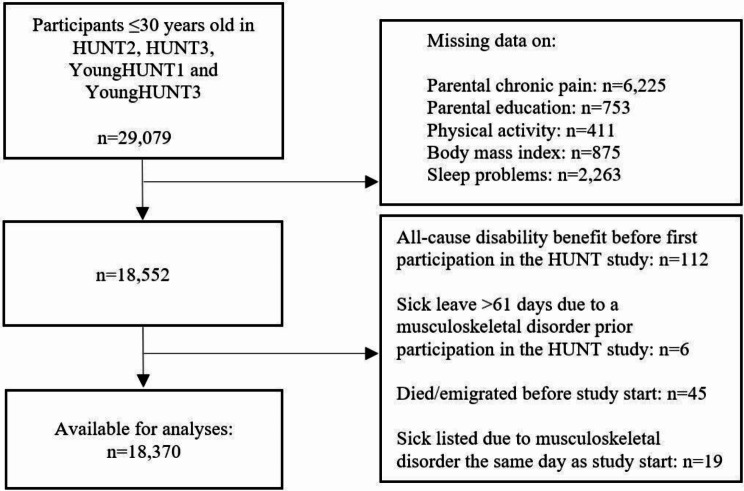
Flowchart for the selection of the study population

A total of 29,079 adolescents and young adults ≤ 30 years participated in either YoungHUNT1 (1995-97), YoungHUNT3 (2006-08), HUNT2 (1995-97), or HUNT3 (2006-08). Of these, 10,527 participants were excluded due to missing data on the exposures or covariates, leaving 11,786 adolescents who participated in YoungHUNT1 or YoungHUNT3, as well as 6,766 young adults ≤ 30 years participating in HUNT2 or HUNT3. Persons with registered all-cause disability benefit (*n* = 112), who had registered sick leave > 61 days due to musculoskeletal disorder the year prior to their first participation (*n* = 6), who died or emigrated (*n* = 45) prior to study start, or participants sick listed due to musculoskeletal disorder the same day as study start (*n* = 19) were excluded. This left 18,370 participants available for statistical analyses (Fig. 1).

### Participants excluded due to missing data

The 10,527 participants excluded due to missing data had a slightly higher proportion of long-term sick leave due to musculoskeletal disorders (37.5% vs. 34.8%) but had largely similar mean age (20.4 years [Standard Deviation (SD) 5.1]) and similar male/female ratio as the included participants.

### Ethics

The study was approved by the Regional Committee for Medical and Health Research Ethics in Central Norway (Ref. no. 230429). The results are presented according to the STROBE statement [31].

### Long-term sick leave in offspring

In the Norwegian benefit scheme, medical benefits usually cover 100% of the wage loss up to one year. The employer pays the first 16 days of the sick leave period, while NAV covers the rest. If the employee has not returned to work after one year, they may apply for more long-term benefits.

Information on registered sick leave was available from 1st January 2000. Sick leave due to musculoskeletal disorders were identified based on the 9th and 10th revisions of International Classification of Diseases (codes M00-M99) and The International Classification of Primary Care (codes L00-L99). Long-term sick leave was defined as a period lasting ≥ 31 consecutive days. The start date of the period was used as the date for the outcome event.

Study entry was set to the date of participation in their first HUNT survey or their 20th birthday if they were younger at participation in HUNT. If both these dates occurred before 1st January 2000, when registry data became available, we used 1st January 2000 as entry date. The participants were followed until a long-term sick-listing of ≥ 31 consecutive days due to a musculoskeletal disorder, emigration, death, granting of a permanent disability benefit (all-cause) or end of follow up 31st December 2021, whichever occurred first.

### Parental characteristics

Information on parental chronic pain was obtained from the earliest possible survey (HUNT2, HUNT3 or HUNT4). Chronic pain was assessed by the question “In the last year, have you had pain and/or stiffness/discomfort/aching in your muscles/joints/limbs that has lasted for at least 3 consecutive months?” (yes/no), with some variation in wording across the surveys. Paternal and maternal pain was combined as participants were classified as (1) “none” (none of the parents reported pain) and (2) “mother or father” (at least one of the parents reported pain). Participants were also placed in the latter category (i.e., “mother or father”) if only one parent reported pain and the other had missing data on this variable.

Data on parental disability benefits were obtained from NAV and coded as (1) “no” if none of the parents were registered with a disability benefit and (2) “yes” if at least one of the parents received disability benefits (all-cause) regardless of the grade and time of attainment.

Information on parental education was provided by Statistics Norway, defined as the highest completed education the year they first participated in the HUNT study and classified as (1) “primary- and upper secondary school” and (2) “university or college”. The latter included any kind of education at university level and was not restricted to a completed degree.

### Offspring lifestyle

Sleep problems in YoungHUNT1, YoungHUNT3 and HUNT2 were assessed by the question “During the last month, have you had any problems falling asleep?” with the response options “never”, “sometimes”, “often”, or “almost every night”. In HUNT3, sleep problems were assessed by the question “How often in the last 3 months have you had difficulty falling asleep at night?” with the response options “never/seldom”, “sometimes” or “several times a week”. Participants in YoungHUNT1, YoungHUNT3 and HUNT2 were classified as having sleep problems “sometimes/often” if they answered “sometimes”, “often”, or “almost every night”, whereas participants in HUNT3 were classified with sleep problems if they answered “sometimes” or “several times a week”.

The questions about leisure time physical activity differed somewhat between the surveys. In YoungHUNT1 and YoungHUNT3, participants were asked “Outside school hours: How many hours a week do you play sports or exercise in your free time so much that you get out of breath or sweat?” with the response options “none”, “about ½ hour”, “about 1 hour”, “about 2–3 hours”, “about 4–6 hours”, or “7 or more hours”. In HUNT2, participants were asked about light (“no sweating/ not out of breath”) and hard (“sweating/out of breath”) leisure time physical activity, both using the question; “How much of your leisure time have you been physically active during the last year (Think of a weekly average for the year. Your commute to work counts as leisure time)?”, with the four response options “0”, “<1”, “1–2”, or “≥3 hours”. In HUNT3, leisure time physical activity per week was reported according to usual frequency (“0”, “<1”, “1”, “2–3”, or “≥4 times per week”), intensity (“no sweat/not out of breath”, “sweat/out of breath”, or “taking it all out”) and duration of each session (“<15”, “15–30”, “31–60”, or “>60 minutes”). For the purpose of assessing effect modification by physical activity while preserving statistical power we constructed a binary variable classifying participants as either (1) “inactive/low activity” (< 3 h light and no hard activity) or (2) “moderate/high activity” (at least ≥ 3 h light and/or < 1 h hard activity).

Body mass index (BMI) was calculated from baseline measurements of weight and height (kg/m^2^). For participants < 18 years at participation, we used age- and sex-adjusted BMI measure based on underlying Lambda-Mu-Sigma curves from the International Obesity Task Force [32].

### Statistical analysis

We used Cox regression to estimate hazard ratio (HR) with 95% confidence interval (CI) for long-term sick leave due to musculoskeletal disorders associated with parental chronic pain, parental disability benefit and parental education. To account for dependency between observations (i.e., siblings), all standard errors were adjusted. Clusters were defined based on parent’s id. Participants with one or both parents having chronic pain were compared with the reference group with parents without chronic pain; participants having parents with disability benefits were compared with participants who had no parents on disability benefits; and participants who had parents with low education were compared with those having parents with high education. We used the same Cox regression models to estimate the joint effect of each parental factor (i.e., chronic pain, disability benefit, and education) combined with either offspring sleep problems or physical activity. To assess possible effect modification we estimated the relative excess risk due to interaction (RERI), which measure interaction on an additive scale [33]. A RERI value > 0 indicates a synergistic effect beyond additivity.

All estimated associations were adjusted for offspring age and sex; independent effects of parental chronic pain and disability benefits were additionally adjusted for parental education; similarly, the joint effects involving parental chronic pain were adjusted for parental education and parental disability benefits; joint effects involving parental disability benefits where adjusted for parental education; joint effects involving parental education were adjusted for parental disability benefits; joint effects involving offspring sleep problems were adjusted for offspring physical activity and BMI; and finally, joint effects involving offspring physical activity were adjusted for offspring sleep problems.

We performed several sensitivity analyses to assess the robustness of the results: (1) we repeated the analyses defining sick leave as i) ≥ 16 days, and ii) ≥ 62 days; (2) we delayed study entry for participants in ongoing higher education lasting at least 2 years until this was completed (as students only have sick leave rights if they have paid employment); (3) we only included parental disability benefits if it was obtained before turning 51 years; and (4) we additionally adjusted for parental chronic pain in the analysis of parental disability benefits.

All analyses were performed using STATA statistical software (version 17) [34].

## Results

Among 18,370 adolescents and young adults, 6,393 (34.8%) experienced long-term sick leave due to a musculoskeletal disorder during a median follow up period of 12.8 years and 248,694 person years. A total of 624 participants (3.4%) were censored during follow up because they received disability benefits, of which 18 were due to musculoskeletal disorders. Table 1 shows descriptive statistics of the study sample according to parental chronic pain, parental disability benefits and parental education. The mean age at participation was 19.1 years (SD 4.7) and 53% were female.

**Table 1:** Characteristics of the study population^a^ (*N* = 18,370), stratified by parental characteristics

	Parental chronic pain		Parental disability benefits		Parental education	
	None	Mother or father	None	Mother or father	University	Primary-/ upper secondary school
Participants, n	4,789	13,581	9,190	9,180	5,709	12,661
Events, long-term sick leave^b^	1,266	5,127	2,499	3,894	1,323	5,070
Age, mean (SD), years	18.3 (4.3)	19.4 (4.8)	18.3 (4.3)	19.9 (5.0)	18.0 (4.0)	19.6 (4.9)
Age categories						
< 15 years	25.0	20.3	25.0	18.1	24.5	20.2
15–19 years	46.6	41.8	46.9	39.2	50.0	39.9
20–24 years	18.5	20.3	17.5	22.1	18.0	20.6
≥ 25 years	9.9	17.6	10.6	20.7	7.5	19.3
Parental chronic pain^c^			64.6	83.3	66.1	77.5
Parental disability	32.0	56.3			34.9	56.7
benefits^d^						
Parental education below	59.6	72.2	59.6	78.3		
university level^e^						
Trouble falling asleep^f^	48.9	50.1	51.2	48.4	53.5	48.1
Moderate/high physical activity^g^	75.4	71.6	74.9	70.3	78.0	70.2
Body mass index, mean (SD), kg/m^2^	23.5 (3.8)	23.1 (3.5)	23.1 (3.4)	23.8 (4.0)	22.7 (3.2)	23.8 (3.9)

*Abbreviations* SD = standard deviation

^a^ Given in % unless other is specified

^b^ Sick leave due to a musculoskeletal disorder at least 31 consecutive days

^c^ Reported chronic pain in either mother or father

^d^ Registered disability benefit in either mother or father

^e^ Highest completed education level among parents

^f^ Any reported degree of problems falling asleep

^g^ ≥3 h light and/or < 1 h hard activity, or any light and ≥ 1 h hard activity

### Independent effect of parental factors

Overall, offspring of parents with chronic pain, who received disability benefits or with low education had an increased risk of long-term sick leave due to a musculoskeletal disorder (Table 2). Compared to the reference group in which none of the parents had chronic pain, offspring of parents with chronic pain had a HR of 1.36 (95% CI 1.27–1.45) for long-term sick leave. Offspring of parents receiving disability benefits had a HR for long term-sick leave of 1.41 (95% CI 1.33–1.48), compared to offspring of parents who did not receive disability benefits. Offspring of parents with low education had a HR of 1.78 (95% CI 1.67–1.90) for long-term sick leave, compared to offspring of parents with high education (Table 2).

**Table 2:** Parental factors and risk of long-term sick leave due to musculoskeletal disorders

	Person- years	Cases	IR	Age-adjusted^a^ HR	Multi-adjusted^b^ HR (95% CI)
Parental chronic pain^c^					
No	66,059	1,266	19.2	1.00	1.00 (Ref.)
Yes	182,635	5,127	28.1	1.44	1.36 (1.27–1.45)
Parental disability benefits^c^					
No	124,361	2,499	20.1	1.00	1.00 (Ref.)
Yes	124,333	3,894	31.3	1.52	1.41 (1.33–1.48)
Parental education					
University	80,031	1,323	16.5	1.00	1.00 (Ref.)
Primary/ upper secondary school	168,664	5,070	30.1	1.78	1.78 (1.67–1.90)

*Abbreviations* CI = confidence interval; HR = hazard ratio; IR = incidence rate per 1000 person-years

^a^ Adjusted for offspring age (years)

^b^ Adjusted for offspring age (years) and sex (man/ woman)

^c^ Also adjusted for parental education level (primary- and upper secondary school/ university or college)

### Joint effects of parental factors and offspring lifestyle factors

**Fig. 2:**
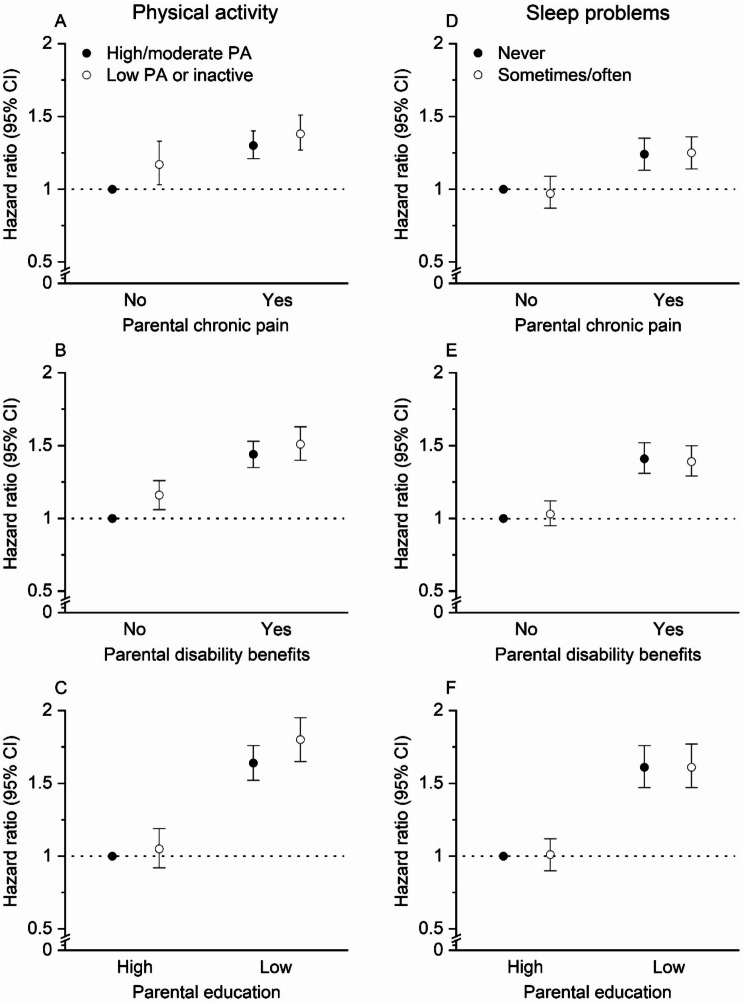
**A-F.** Joint effect of parental chronic pain, parental disability benefits, and parental education with offspring sleep problems and offspring physical activity level on risk of long-term sick leave (≥ 31 days) among offspring due to musculoskeletal disorders. All estimates were adjusted for offspring age and offspring sex. In addition, **A**-**C** were adjusted for offspring sleep problems, **D**-**F** were adjusted for offspring physical activity and BMI, **A** and **D** were adjusted for parental education and parental disability benefits, **B** and **E** were adjusted for parental education, and **C** and **F** were adjusted for parental disability benefits

There was no statistical evidence of a synergistic effect between parental factors and offspring lifestyle factors on the risk of long-term sick leave due to musculoskeletal disorders (RERI estimates ranging from − 0.09 to 0.11) (Fig. 2A-F, and Additional file Table 7). Compared to highly physically active offspring of parents without chronic pain, offspring of parents with chronic pain had HRs of 1.30 (95% CI 1.21–1.40) if they were active and 1.38 (95% CI 1.27–1.51) if they were inactive. Offspring who were inactive and did not have parents with chronic pain had a HR of 1.17 (95% CI 1.03–1.33), compared to the same reference category (Additional file Table 6). Similar trends were observed for all the joint effect combinations (Fig. 2) and are presented in detail in Additional file Table 6.

### Sensitivity analyses

The sensitivity analyses had minor impact on the results of separate effects. First, using a sick-leave length of ≥ 16 days gave similar HRs compared to the main analyses, whereas using ≥ 62 days gave similar or slightly stronger but somewhat less precise HRs (Additional file Tables 1 and 2, respectively). Delaying study entry for those who completed at least 2 years of higher education slightly attenuated the HRs of sick leave, especially for parental education (Additional file Table 3). Using an upper age limit of 50 years for parental disability benefit attenuated only the HR of sick leave for parental disability benefits (Additional file Table 4). Conducting the same four sensitivity analyses on the joint effect estimates did not impact the results (data not shown). Finally, the association between parental disability benefits and risk of sick leave was slightly attenuated after adjusting for parental pain (Additional file Table 5).

## Discussion

This prospective family-linkage study indicates that parental chronic pain, disability benefits, and low education increase the risk of long-term sick leave due to a musculoskeletal disorder in offspring. These associations were not modified by offspring sleep problems or physical activity.

Our results are in line with previous studies reporting that parental chronic pain have a strong influence on chronic pain in offspring [35, 36]. A previous study from the HUNT population found an increased risk of receiving all-cause medical benefits among participants that had parents receiving medical benefits or parents with low education [37]. The parental factors were obtained while the participants were adolescents, and likely to have a shared environment with their parents. In contrast, we did not restrict parental factors to a specific age or period. However, we conducted a sensitivity analysis where we restricted parental age to ≤ 50 years when receiving disability benefit, but this had negligible influence on the results.

Both sleep problems and physical inactivity have been related to increased risk of long-term sick leave and disability benefits due to musculoskeletal disorders [18–28], but no previous study has examined the joint effects of parental factors and offspring lifestyle factors on risk of long-term sick leave due to musculoskeletal disorders. However, previous studies have also not found offspring physical activity to modify the intergenerational association in chronic widespread pain [36] nor the association between parental chronic pain and activity-limiting chronic musculoskeletal pain in the offspring [35]. In contrast to the current study, both previous studies examined an adult offspring population that may be less likely to share environmental and lifestyle factors with their parents [35, 36].

One third of our participants got sick listed at least 31 days due to a musculoskeletal disorder during follow up regardless of exposure status, underscoring that long-term medical benefits are common among young adults. Thus, a small reduction in risk can be of great importance for many people. As previous studies have focused on the influence of single exposure variables [18, 19, 26], our study is the first to examine the joint effects between parental factors and individual lifestyle factors. In agreement with our findings, the lack of strong modifying effects of lifestyle factors has been demonstrated for mortality, where health risk behaviors at an individual level did not affect the association between socioeconomic factors and all-cause mortality [38, 39]. Along with findings from our study, this suggest that preventive efforts should move beyond a focus on isolated health behaviors (e.g., physical activity) to consider the impact of clustered patterns of risk associated with social context [40]. This means shifting focus from individual factors to familial and societal factors such as social inequality [38, 40].

### Strengths and limitations

The strengths of this study include the large study population and the intergenerational, prospective design with long follow-up using nationwide registries. However, some limitations should be considered: First, we used self-reported data on sleep and physical activity, which is prone to misclassification and recall bias. The sleep questions did not address more severe conditions like insomnia. Previous studies have indicated a dose-response association between number of insomnia symptoms and risk of chronic pain [41, 42], and our study may therefore underestimate the role of insomnia. Furthermore, the questions on physical activity varied between the HUNT surveys. However, the information is likely valid for classifying participants into broad categories of physical activity [43]. Second, we could not include complete parent-offspring trios since this would substantially reduce the number of participants available for analysis. To accommodate this, we used strict criteria to classify offspring into expected beneficial categories of the parental characteristics, which could rather underestimate the true effect. Third, it is difficult to disentangle the causal relations between BMI, sleep, and physical activity due to their possible bidirectionality. Hence, BMI was not included as an adjustment variable in the analyses of physical activity and risk of sick leave, since BMI may partly mediate this association. On the contrary, since overweight, and in particular obesity, are associated with increased risk of sleep disorders [44], we adjusted for BMI in the analyses of sleep problems and risk of sick leave. Finally, we cannot rule out residual confounding due to unknown and poorly measured factors.

## Conclusions

Parental chronic pain, disability benefits and low education were associated with an increased risk of long-term sick leave due to musculoskeletal disorders among young adult offspring. The effects of the parental factors were not modified by offspring sleep problems or physical activity level, suggesting that efforts beyond individual lifestyle factors might be important as preventive measures.

## Electronic supplementary material

Below is the link to the electronic supplementary material.

Supplementary Material 1

## Data Availability

The data that support the findings of this study are available from HUNT (https://www.ntnu.edu/hunt/), NAV (https://www.nav.no/) and Statistics Norway (https://www.ssb.no/en) but restrictions apply to the availability of these data, which were used under license for the current study, and so are not publicly available. Enquiries about the data materials used in this study can be sent by mail to lene.aasdahl@ntnu.no.

## Abbreviations

BMI: Body mass index
CI: Confidence interval
HR: Hazard ratio
HUNT: The Trøndelag Health Study
IR: Incidence rate per 1000 person-years
NAV: The Norwegian labour and welfare administration
RERI: The relative excess risk due to interaction
SD: Standard deviation
STROBE: Strengthening the reporting of observational studies in epidemiology
