# Supplementary material for: The effect of parental pain, disability benefits and education on risk of long-term sick leave due to musculoskeletal disorders and the modifying effect of sleep and physical activity: the HUNT study
Source: BMC Public Health. 2024 Sep 27;24:2617. doi: 10.1186/s12889-024-20071-1 (PMC11438124; doi:10.1186/s12889-024-20071-1)
Supplement: Supplementary file 1 — Supplementary Material 1 [file 12889_2024_20071_MOESM1_ESM.docx]

**Additional file**

**Table 1** Separate effects of parental chronic pain, parental disability benefits, and parental education on the risk of sick leave ≥16 days among offspring due to musculoskeletal disorder.

**Table 2** Separate effects of parental chronic pain, parental disability benefits, and parental education on the risk of sick leave ≥62 days among offspring due to musculoskeletal disorder.

**Table 3** Separate effects of parental chronic pain, parental disability benefits, and parental education on the risk of sick leave ≥31 days among offspring due to musculoskeletal disorder, with postponed follow-up for those in ongoing higher education.

**Table 4** Separate effects of parental chronic pain, parental disability benefits, and parental education on the risk of sick leave ≥31 days among offspring due to musculoskeletal disorder, with an age limit of 50 years for parental disability benefits.

**Table 5** Separate effects of parental chronic pain, parental disability benefits, and parental education on the risk of sick leave ≥31 days among offspring due to musculoskeletal disorder, additionally adjusted for parental chronic pain.

**Table 6** Joint effect of parental chronic pain, disability benefits, and educational with offspring sleep problems and physical activity level, on the risk of sick leave ≥31 days among offspring due to musculoskeletal disorders.

**Table 7** Relative excess risk due to interaction (RERI), estimated from the adjusted Hazard Ratios of the joint effect of parental factors and offspring lifestyle factors.

**Table 1.** Separate effects of parental chronic pain, parental disability benefits, and parental education on the risk of sick leave ≥16 days among offspring due to musculoskeletal disorder.

|  | Person- | Cases | IR | Age-adjusted^a^ | Multi-adjusted^b^ |
| --- | --- | --- | --- | --- | --- |
|  | years |  |  | HR | HR (95% CI) |
| Parental chronic pain^c^ |  |  |  |  |  |
| No | 62,250 | 1,602 | 25.7 | 1.00 (Ref.) | 1.00 (Ref.) |
| Yes | 168,022 | 6,226 | 36.2 | 1.43 | 1.34 (1.27-1.42) |
| Parental disability benefits^c^ |  |  |  |  |  |
| No | 116,663 | 3,164 | 27.1 | 1.00 (Ref.) | 1.00 (Ref.) |
| Yes | 113,309 | 4,664 | 41.1 | 1.49 | 1.38 (1.31-1.45) |
| Parental education |  |  |  |  |  |
| University | 76,077 | 1,671 | 22.0 | 1.00 (Ref.) | 1.00 (Ref.) |
| Primary/ upper secondary | 154,195 | 6,157 | 40.0 | 1.80 | 1.80 (1.70-1.91) |
| school | | | | | |

Abbreviations: CI = Confidence interval; HR = hazard ratio; IR= incidence rate per 1000 person-years
^a^ Adjusted for offspring age (years)
^b^ Adjusted for offspring age (years) and offspring sex (man/ woman)
^c^ Also adjusted for parental education level (primary- and upper secondary school/ university or college)

**Table 2.** Separate effects of parental chronic pain, parental disability benefits, and parental education on the risk of sick leave ≥62 days among offspring due to musculoskeletal disorder.

|  | Person- | Cases | IR | Age-adjusted^a^ | Multi-adjusted^b^ |
| --- | --- | --- | --- | --- | --- |
|  | years |  |  | HR | HR (95% CI) |
| Parental chronic pain^c^ |  |  |  |  |  |
| No | 70,087 | 877 | 12.5 | 1.00 (Ref.) | 1.00 (Ref.) |
| Yes | 198,877 | 3,800 | 19.1 | 1.49 | 1.40 (1.29-1.51) |
| Parental disability benefits^c^ |  |  |  |  |  |
| No | 132,630 | 1,725 | 13.0 | 1.00 (Ref.) | 1.00 (Ref.) |
| Yes | 136,333 | 2,952 | 21.7 | 1.60 | 1.48 (1.39-1.58) |
| Parental education |  |  |  |  |  |
| University | 84,392 | 905 | 10.7 | 1.00 (Ref.) | 1.00 (Ref.) |
| Primary/ upper secondary | 184,571 | 3,772 | 20.4 | 1.84 | 1.84 (1.70-1.98) |
| school | | | | | |

Abbreviations: CI = Confidence interval; HR = hazard ratio; IR= incidence rate per 1000 person-years
^a^ Adjusted for offspring age (years)
^b^ Adjusted for offspring age (years) and offspring sex (man/ woman)
^c^ Also adjusted for parental education level (primary- and upper secondary school/ university or college)

**Table 3.** Separate effects of parental chronic pain, parental disability benefits, and parental education on the risk of sick leave ≥31 days among offspring due to musculoskeletal disorder, with postponed follow-up for those in ongoing higher education.

|  | Person- | Cases | IR | Age-adjusted^a^ | Multi-adjusted^b^ |
| --- | --- | --- | --- | --- | --- |
|  | years |  |  | HR | HR (95% CI) |
| Parental chronic pain^c^ |  |  |  |  |  |
| No | 54,878 | 1,187 | 21.6 | 1.00 (Ref.) | 1.00 (Ref.) |
| Yes | 159,826 | 4,831 | 30.2 | 1.39 | 1.32 (1.24-1.41) |
| Parental disability benefits^c^ |  |  |  |  |  |
| No | 103,471 | 2,306 | 22.3 | 1.00 (Ref.) | 1.00 (Ref.) |
| Yes | 111,232 | 3,712 | 33.4 | 1.48 | 1.39 (1.32-1.47) |
| Parental education |  |  |  |  |  |
| University | 63,646 | 1,202 | 18.9 | 1.00 (Ref.) | 1.00 (Ref.) |
| Primary/ upper secondary | 151,057 | 4,816 | 31.9 | 1.69 | 1.69 (1.58-1.80) |
| school | | | | | |

Abbreviations: CI = Confidence interval; HR = hazard ratio; IR= incidence rate per 1000 person-years
^a^ Adjusted for offspring age (years)
^b^ Adjusted for offspring age (years) and offspring sex (man/ woman)
^c^ Also adjusted for parental education level (primary- and upper secondary school/ university or college)

**Table 4.** Separate effects of parental chronic pain, parental disability benefits, and parental education on the risk of sick leave ≥31 days among offspring due to musculoskeletal disorder, with an age limit of 50 years for parental disability benefits.

|  | Person- | Cases | IR | Age-adjusted^a^ | Multi-adjusted^b^ |
| --- | --- | --- | --- | --- | --- |
|  | years |  |  | HR | HR (95% CI) |
| Parental chronic pain^c^ |  |  |  |  |  |
| No | 66,059 | 1,266 | 19.2 | 1.00 (Ref.) | 1.00 (Ref.) |
| Yes | 182,621 | 5,127 | 28.1 | 1.44 | 1.36 (1.27-1.45) |
| Parental disability benefits^c^ |  |  |  |  |  |
| No | 219,973 | 5,408 | 24.6 | 1.00 (Ref.) | 1.00 (Ref.) |
| Yes | 28,707 | 985 | 34.3 | 1.43 | 1.32 (1.22-1.42) |
| Parental education |  |  |  |  |  |
| University | 80,031 | 1,323 | 16.5 | 1.00 (Ref.) | 1.00 (Ref.) |
| Primary/ upper secondary | 168,649 | 5,070 | 30.1 | 1.78 | 1.78 (1.67-1.90) |
| school | | | | | |

Abbreviations: CI = Confidence interval; HR = hazard ratio; IR= incidence rate per 1000 person-years
^a^ Adjusted for offspring age (years)
^b^ Adjusted for offspring age (years) and offspring sex (man/ woman)
^c^ Also adjusted for parental education level (primary- and upper secondary school/ university or college)

|  | Person-  years | Cases | IR | Age-adjusted^a^  HR | Multi-adjusted^b^  HR (95% CI) |
| --- | --- | --- | --- | --- | --- |
| Parental chronic pain^c^ |  |  |  |  |  |
| No | 66,059 | 1,266 | 19.2 | 1.00 | 1.00 (Ref.) |
| Yes | 182,635 | 5,127 | 28.1 | 1.44 | 1.36 (1.27-1.45) |
| Parental disability benefits^d^ |  |  |  |  |  |
| No | 124,361 | 2,499 | 20.1 | 1.00 | 1.00 (Ref.) |
| Yes | 124,333 | 3,894 | 31.3 | 1.52 | 1.35 (1.28-1.43) |
| Parental education |  |  |  |  |  |
| University | 80,031 | 1,323 | 16.5 | 1.00 | 1.00 (Ref.) |
| Primary/ upper secondary  school | 168,664 | 5,070 | 30.1 | 1.78 | 1.78 (1.67-1.90) |
|  | | | | | |

**Table 5.** Separate effects of parental chronic pain, parental disability benefits, and parental education on the risk of sick leave ≥31 days among offspring due to musculoskeletal disorder, additionally adjusted for parental chronic pain.

Abbreviations: CI = confidence interval; HR = hazard ratio; IR = incidence rate per 1000 person-years
^a^ Adjusted for offspring age (years)

^b^ Adjusted for offspring age (years) and sex (man/ woman)

^c^ Also adjusted for parental education level (primary- and upper secondary school/ university or college)
^d^ Also adjusted for parental education level (primary- and upper secondary school/ university or college) and parental chronic pain (yes/ no)

|  | Offspring sleep problems^a^ | | | | |  | Offspring physical activity^b^ | | | | |
| --- | --- | --- | --- | --- | --- | --- | --- | --- | --- | --- | --- |
|  | Never | |  | Sometimes/Often | |  | High/Moderate | |  | Low/inactive | |
|  | Cases/ | Adjusted |  | Cases/ | Adjusted |  | Cases/ | Adjusted |  | Cases/ | Adjusted |
|  | Person-years | HR (95% CI) |  | Person-years | HR (95% CI) |  | Person-years | HR (95% CI) |  | Person-years | HR (95% CI) |
| Parental |  |  |  |  |  |  |  |  |  |  |  |
| chronic pain^c^ |  |  |  |  |  |  |  |  |  |  |  |
| No | 672/34,543 | 1.00 (Ref.) |  | 594/31,517 | 0.97 (0.87-1.09) |  | 918/50,420 | 1.00 (Ref.) |  | 348/15,639 | 1.17 (1.03-1.33) |
| Yes | 2,636/93,339 | 1.24 (1.13-1.35) |  | 2,491/89,296 | 1.25 (1.14-1.36) |  | 3,601/132,163 | 1.30 (1.21-1.40) |  | 1,526/50,472 | 1.38 (1.27-1.51) |
| Parental |  |  |  |  |  |  |  |  |  |  |  |
| disability |  |  |  |  |  |  |  |  |  |  |  |
| benefits^d^ |  |  |  |  |  |  |  |  |  |  |  |
| No | 1,244/62,503 | 1.00 (Ref.) |  | 1,255/61,858 | 1.03 (0.95-1.12) |  | 1,796/93,712 | 1.00 (Ref.) |  | 703/30,649 | 1.16 (1.06-1.26) |
| Yes | 2,064/65,378 | 1.41 (1.31-1.52) |  | 1,830/58,955 | 1.39 (1.29-1.50) |  | 2,723/88,871 | 1.44 (1.35-1.53) |  | 1,171/35,462 | 1.51 (1.40-1.63) |
| Parental |  |  |  |  |  |  |  |  |  |  |  |
| education^e^ |  |  |  |  |  |  |  |  |  |  |  |
| University | 624/38,186 | 1.00 (Ref.) |  | 699/41,845 | 1.01 (0.90-1.12) |  | 1,010/62,152 | 1.00 (Ref.) |  | 313/17,878 | 1.05 (0.92-1.19) |
| Primary/ | 2,684/89,695 | 1.61 (1.47-1.76) |  | 2,386/78,969 | 1.61 (1.47-1.77) |  | 3,509/120,431 | 1.64 (1.52-1.76) |  | 1,561/48,233 | 1.80 (1.65-1.95) |
| upper |  |  |  |  |  |  |  |  |  |  |  |
| secondary |  |  |  |  |  |  |  |  |  |  |  |
| school |  |  |  |  |  |  |  |  |  |  |  |

**Table 6.** Joint effect of parental chronic pain, disability benefits, and education with offspring sleep problems and physical activity level, on the risk of sick leave ≥31 days among offspring due to musculoskeletal disorders.

Abbreviations: CI = confidence interval; HR = hazard ratio
^a^ Adjusted for offspring physical activity level (high-moderate/ low-inactive) and offspring body mass index (kg/m^2^)
^b^ Adjusted for offspring sleep problems (never/ sometimes-often)
^c^ Adjusted for offspring age (years), offspring sex (man/ woman), parental education (primary- and upper secondary school/ university or college), and parental disability benefits (yes/ no)
^d^ Adjusted for offspring age (years), offspring sex (man/ woman), and parental education (primary- and upper secondary school/ university or college)
^e^ Adjusted for offspring age (years), offspring sex (man/ woman), and parental disability benefits (yes/ no)

**Table 7.** Relative excess risk due to interaction (RERI), estimated from the adjusted Hazard Ratios of the joint effect of parental factors and offspring lifestyle factors.

| Joint effect | |  |  | RERI (95% CI) | |
| --- | --- | --- | --- | --- | --- |
| Offspring sleep problems and parental chronic pain | |  |  | 0.04 | (-0.09 to 0.17) |
| Offspring sleep problems and parental disability benefits | |  | - | 0.05 | (-0.18 to 0.07) |
| Offspring sleep problems and parental education | |  | - | 0.00 | (-0.14 to 0.14) |
| Offspring physical activity and parental chronic pain | |  | - | 0.09 | (-0.25 to 0.08) |
| Offspring physical activity and parental disability benefits | |  | - | 0.08 | (-0.23 to 0.06) |
| Offspring physical activity and parental education | |  |  | 0.11 | (-0.06 to 0.28) |
|  |  | | | | |

Abbreviations: CI = confidence interval; RERI= relative excess risk due to interaction
